# Supplementary material for: Metabolic and Transcriptional Analysis Reveals Flavonoid Involvement in the Drought Stress Response of Mulberry Leaves
Source: Int J Mol Sci. 2024 Jul 6;25(13):7417. doi: 10.3390/ijms25137417 (PMC11242228; doi:10.3390/ijms25137417)
Supplement: Supplementary file 1 [file ijms-25-07417-s001.zip › Revised supporting information.pdf]

Supporting Information for

Metabolic and Transcriptional Analysis Reveals Flavonoid Involvement in the Drought Stress Response of Mulberry Leaves

Guo Chen[#1], Dong Li[#1], Pei Yao[1], Fengyao Chen[1], Jianglian Yuan[1], Bi Ma[1], Zhen Yang[1], Biyue Ding[2] and Ningjia He*[1]

[1]State Key Laboratory of Resource Insects, Institute of Sericulture and Systems Biology, Southwest University, Chongqing 400715, China

[2]Key Laboratory of Agricultural Biosafety and Green Production of Upper Yangtze River (Ministry of Education), Academy of Agricultural Sciences, Southwest University, Chongqing 400715, China

Guo Chen: q542523966@163.com

Dong Li: lidong203@swu.edu.cn

Pei Yao: wanmbbmmdd@email.swu.edu.cn

Fengyao Chen: cfy0127@email.swu.edu.cn

Jianglian Yuan: yuanjiangl@swu.edu.cn

Bi Ma: mbzls@swu.edu.cn

Zhen Yang: yangzhen1246305364@163.com

Biyue Ding: dingbiyue302@swu.edu.cn

Ningjia He: hejia@swu.edu.cn

[#]These authors contributed equally to this work

*Corresponding author: Ningjia He, State Key Laboratory of Resource Insects, Institute of Sericulture and Systems Biology, Southwest University, Chongqing 400715, China; Tel: +86-023-68250797; Fax: +86-023-68251128; E-mail: hejia@swu.edu.cn.

This file includes:

Figure S1-S3, Table S3-S5

Supplementary Materials:

Figure S1. Metabolic and transcriptional analysis of *M. alba* var. *pendula* leaves under drought stress.

Figure S2. Bioinformatic analysis of *MaUGTs*.

Figure S3. Bioinformatic analysis of *MaFLSs*.

Table S1. Checklist of flavonoids measured in this study and recommendations for LC-MS/MS. Table is on a separate sheet.

Table S2. Metabolic profiling of flavonoids in *M. alba* var. *pendula* leaves under drought stress. Table is on a separate sheet.

Table S3. Statistical results of RNA-seq assembly data.

Table S4. Details of significantly changed genes involved in flavonoid biosynthesis in this study.

Table S5. List of primer sequences used in this study.

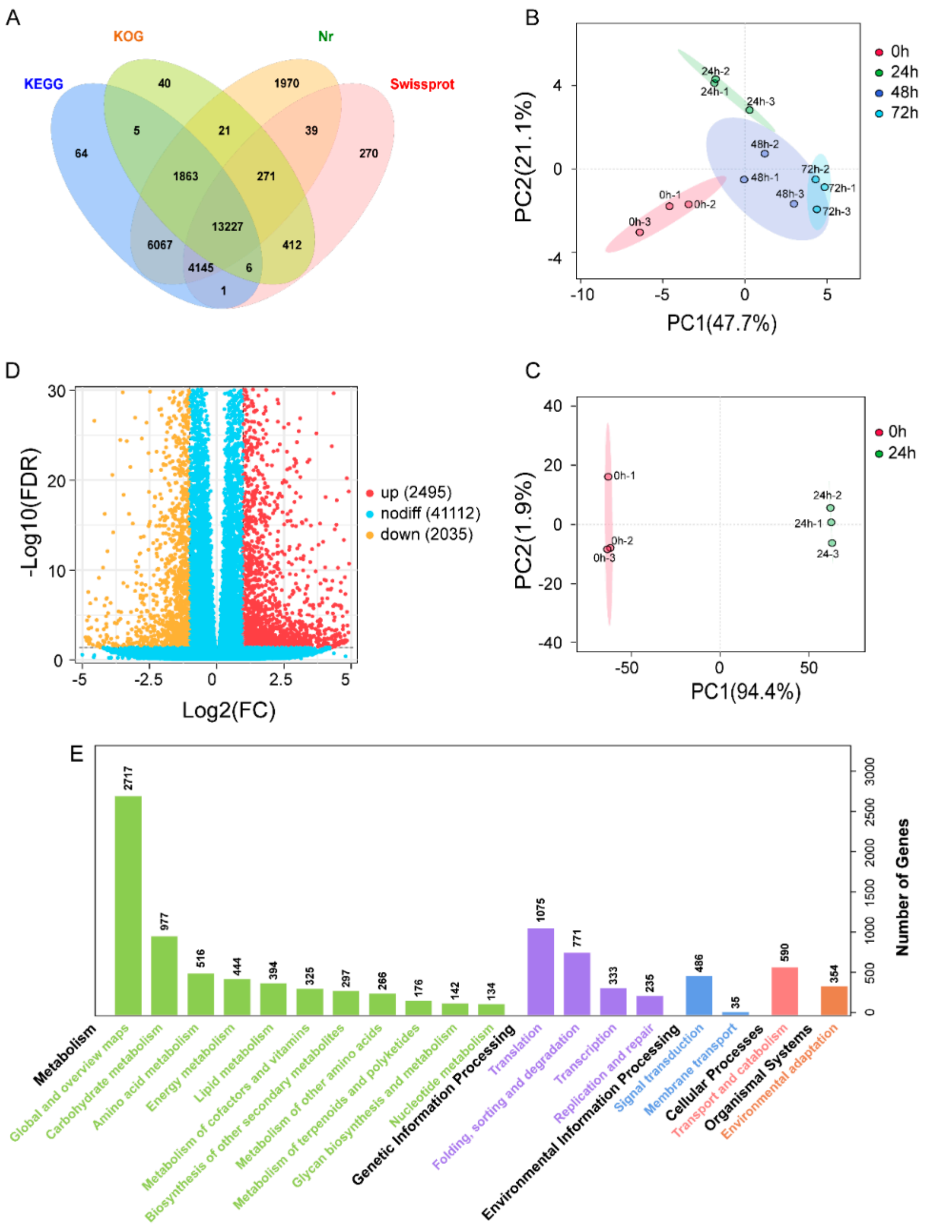

Figure S1. Metabolic and transcriptional analysis of *M. alba* var. *pendula* leaves under drought stress. (**A**) Venn Diagram showing annotation genes across four databases. (**B**) Principal component analysis (PCA) of the metabolome. (**C**) PCA of the transcriptome. (**D**) Volcano plot displaying differentially expressed genes (DEGs) identified through transcriptome analysis. (**E**) KEGG pathways of DEGs in mulberry leaves under drought conditions.

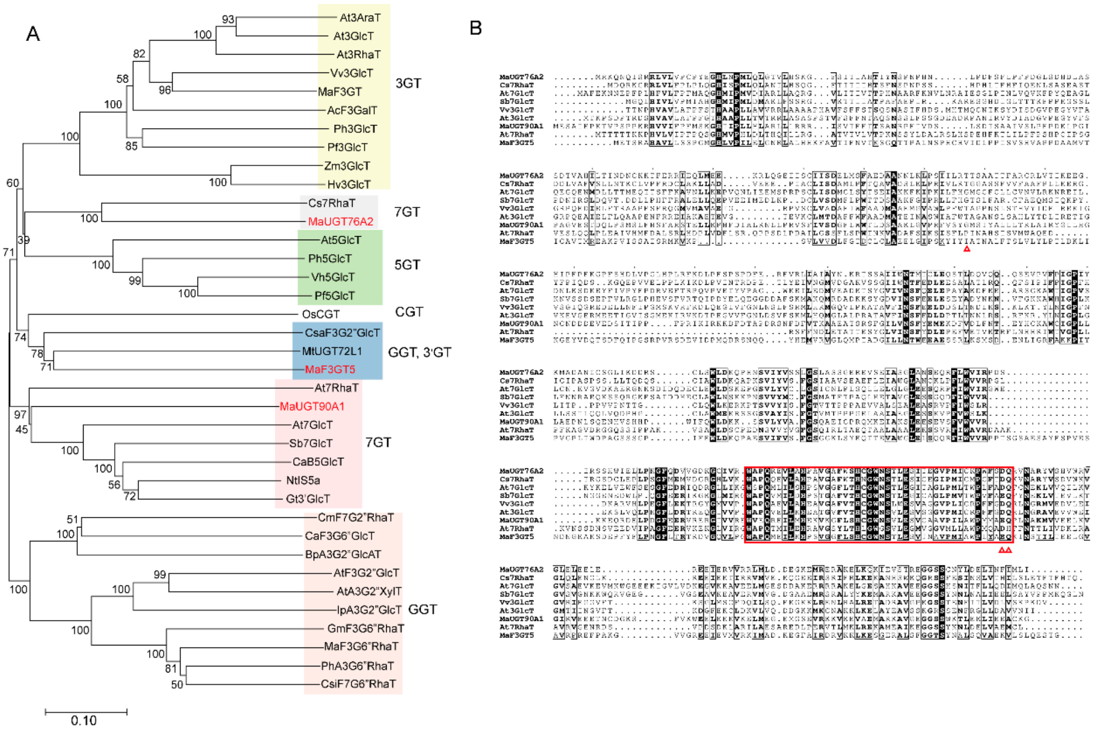

Figure S2. Bioinformatic analysis of *MaUGTs*. (**A**) Non-rooted molecular phylogenetic tree of flavonoid related UDP-glycosyltransferase. Three candidate *MaUGTs* genes analyzed in this study are shown in red color. The GenBank accession numbers for the sequences are shown in parentheses: *At3GlcT* (NM_121711), *At3AraT* (NM_121709), *At3RhaT* (NM_102790), *Vv3GlcT* (AF000371), *Ph3GalT* (AF316552), *AcF3GalT* (GU079683), *Ph3GlcT* (AB027454), *Pf3GlcT* (AB002818), *Zm3GlcT* (X13501), *Hv3GlcT* (X15694), *At5GlcT* (NM_117485), *Ph5GlcT* (AB027455), *Vh5GlcT* (AB013598), *Pf5GlcT* (AB013596), *OsCGT* (FM179712), *CsaF3G2"GlcT* (HE793682), *MtUGT72L1* (EU434684), *Cs7RhaT* (KDO69246), *At7RhaT* (NM_100480), *At7GlcT* (NM_129234), *Sb7GlcT* (AB031274), *NtIS5a* (AF346431), *DbB5GlcT* (Y18871), *Gt3'GlcT* (AB076697), *CmF7G2"RhaT* (AY048882), *CaF3G6"GlcT* (AB443870), *BpA3G2"GlcAT* (AB190262), *CsiF7G6"RhaT* (DQ119035), *PhA3G6"RhaT* (Z25802), *GmF3G6"RhaT* (NP_001275524), *IpA3G2"GlcT* (AB192315), *AtF3G2"GlcT* (BT020440), *AtA3G2"XylT* (NM_124785) , and *MaF3G6˝RhaT* (KT324624). (**B**) Alignment of the amino acid sequences of three MaUGTs and other UGT proteins. The PSPG box conserved motif of UGT is presented in the red box. The glycosylation site is indicated in red triangles.

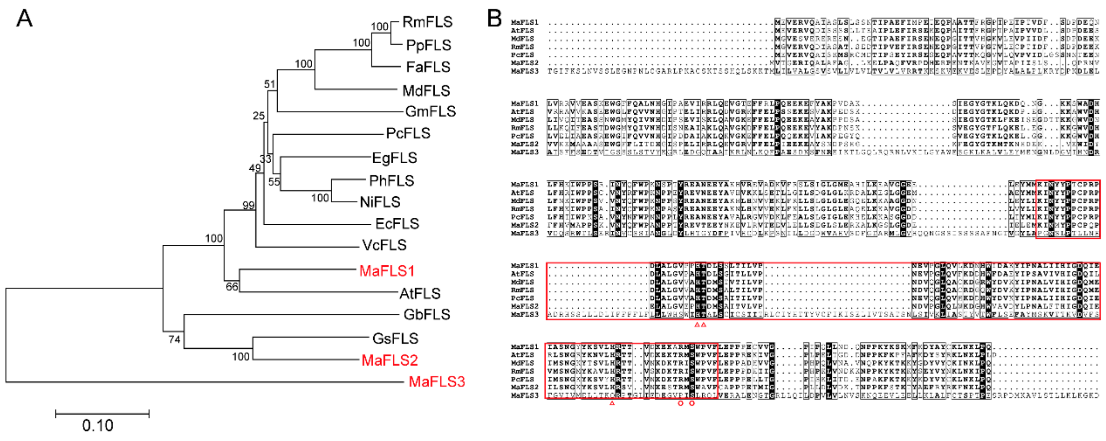

Figure S3. Bioinformatic analysis of *MaFLSs*. (**A**) Phylogenetic analysis of flavonol synthase (FLS). The phylogenetic tree was constructed using the MEGA7 software with a neighbor-joining method. Sequence data from this article can be found in the NCBI database under the following accession numbers: *VcFLS* (AKJ87100), *EcFLS* (ALP48589), *PcFLS* (AY230249), *EgFLS* (AY230249), *MdFLS* (AAD26261), *PhFLS* (Q07512), *FaFLS* (AAZ78661), *RmFLS* (AJP36706), *GmFLS* (NP001237419), *GsFLS* (NP001237419), *AtFLS* (AED91333), *PpFLS* (AJO70134), *GbFLS* (GQ994432), *NiFLS* (AB078512), *MaFLS1* (XM_010098126), *MaFLS2* (XP_010091357), and *MaFLS3* (UTN00788). (**B**) Alignment of the amino acid sequences of the MaFLS1-S3 and other FLS proteins. The typical of conserved 2-oxoglutarate-Fe (II) oxygenase domain is presented in the red box. The Fe^{2+}-binding sites and oxoglutarate binding sites are indicated by red triangles and red ellipses, respectively.

Table S3. Statistical results of RNA-seq assembly data.

Genes number	GC percentage	N50 number	N50 length	Max length	Min length	Average length	Total assembled bases
45642	40.4454	7996	2292	16569	201	1234	56354744

Table S4. Details of significantly changed genes involved in flavonoid biosynthesis in this study.

Gene ID	FPKM[a]		Fold change	Symbol	Putative annotation[b]
	C	D			
Unigene0023038	10.82	25.15	2.32	*PAL1*	Phenylalanine ammonia lyase
Unigene0039394	101.63	235.33	2.32	*PAL2*	Phenylalanine ammonium lyase
Unigene0022114	3.8	6.83	1.80	*PAL3*	Phenylalanine ammonia lyase
Unigene0039210	18.87	30.42	1.61	*4CL1*	4-coumarate--CoA ligase 1
Unigene0020203	4.97	7.23	1.45	*4CL2*	4-coumarate--CoA ligase 1
Unigene0042667	20.98	164.28	7.83	*CHS1*	Chalcone synthase
Unigene0042666	2.25	4.65	2.07	*CHS2*	Chalcone synthase
Unigene0008170	1.25	0.41	0.33	*CHS3*	Chalcone synthase
Unigene0029681	49.98	85.59	1.71	*F3H*	Flavone 3-hydroxylase
Unigene0046320	47.38	114.49	2.42	*F3'H1*	Flavone 3'-hydroxylase
Unigene0046319	5.28	16.23	3.07	*F3'H2*	Flavone 3'-hydroxylase
Unigene0000347	31.78	99.27	3.12	*FLS1*	Flavonol synthase/flavanone 3-hydroxylase
Unigene0014572	2.41	0.09	0.04	*FLS2*	Flavonol synthase/flavanone 3-hydroxylase
Unigene0042252	11.02	5.49	0.50	*FLS3*	Flavonol synthase/flavanone 3-hydroxylase
Unigene0046274	15.85	61.33	3.87	*F3GT*	Anthocyanidin 3-O-glucosyltransferase
Unigene0033543	7.26	71.72	9.88	*F3GT5*	Anthocyanidin 3-O-glucosyltransferase 5
Unigene0015859	1.11	4.98	4.49	*UGT76A2*	UDP-glucose glucosyltransferase 76A2
Unigene0038284	5.5	13.72	2.49	*UGT90A1*	UDP-glucose glucosyltransferase 90A1

[a]Represents average of three replicates of normalized FPKM (reads per kb per million reads) values.

[b]Putative annotations according to BlastX queries against NCBI non-redundant (nr) protein database.

Table S5. List of primer sequences used in this study.

Gategory	Gene	Primer sequences	
		Forward (5' to 3')	Reverse (3' to 5')
Sequencing	*MaFLS1*	ATGGAGGTTGAGAGAGTTCAAG	TCACTGGG GAAGCTTGTTGAG
	MaF3GT5	ATGGGCCACCTAGTTCCGAT	TCAGCCAGTGCT TTCTTTTAAGCT
	MaUGT76A2	ATGCCGGACGGCTTATCC	TCATGCCAAGAAACCAAATTGGTC
	MaUGT90A1	ATGGAATCGGCTATCGAGCC	TTAATAATTACTCGCATGATCTACT
qRT-PCR	*MaPAL1*	CCGGACCGACTTCTTCGAGC	GAATTCGGGCATCCCGAGCA
	MaPAL2	GCAATGGAACAGAATCATGTCACAC	TCTAATGCCTGAGTAGCCTTGGAG
	MaPAL3	TCACCACCGGCTTTGGTTCC	CCGGAGGCGGTTATGGTTCC
	MaC4H	AACCTCCCTTGTAGCTCGTCCTG	CCATACCAGCAGTACCACCAATTCC
	Ma4CL1	CCGAAGGGGGTGATGCTGAC	ATCACGATCGGCGGCACAAT
	Ma4CL2	CATCTCGGAGCCGTCACCAC	TCGTGCCCGAAGAATACGGC
	MaCHS1	TCACACCGATCGGCATCAGC	CCTGCTTCAGCCCCAGCTTT
	MaCHS2	GGCAACCTCCGTCCAAGAAATC	TTGTGCTCGCTGTTGGTGATTC
	MaCHI	GAGATAGCCACGTTCCCGCC	TCCACTTACCGGCAAGCCAC
	MaFLS1	CCTCCCTCTCGCATCAACTACC	AACACCTTATCCGCCACCTCTC
	MaFLS2	TGTCTTGGGCGGTGTTTTGTG	ACTTTGGCGGGTTTGTCTTGTC
	MaF3H	ATCGCGGTGACGGAGGAGTA	GCGCTTCAGGCCAAGAGTGA
	MaF3'H	CGTAATCGGGAACCTGCCCC	TGTGCTTGGCTCCGGAGTTG
	MaF3GT	CATCGTCCGTCGCCTAGCTG	GAACAGCTCGATGCGCTCCT
	MaF3GT5	CTGCCACGTCATCGACCTCC	TCCTCGGCCAGACACAGACA
	MaUGT76A2	TGGAATTGGAGGAGTTGGAGAGAG	AGACGAACCACCTTCCCTTGTAG
	MaUGT90A1	CACTCCTTCGCCCGATCCAC	GCCCATAGCGGATTGCGAGT
	MaF3G6''RT	TAGTCCACTCCGGGTGGGTG	ACCTCCACCCCAGCCTTCAT
	MaActin	GCATGAAGATCAAGGTGGTG	CATCTGCTGGAAGGTGCTAA
